# Bone Marrow Recovery and Subsequent Chemotherapy Following Radiolabeled Anti-Prostate-Specific Membrane Antigen Monoclonal Antibody J591 in Men with Metastatic Castration-Resistant Prostate Cancer

**DOI:** 10.3389/fonc.2013.00214

**Published:** 2013-08-26

**Authors:** Scott T. Tagawa, Naveed H. Akhtar, Anastasia Nikolopoulou, Gurveen Kaur, Brian Robinson, Renee Kahn, Shankar Vallabhajosula, Stanley J. Goldsmith, David M. Nanus, Neil H. Bander

**Affiliations:** ^1^Department of Medicine, Division of Hematology and Medical Oncology, Weill Cornell Medical College, New York, NY, USA; ^2^Department of Urology, Weill Cornell Medical College, New York, NY, USA; ^3^Department of Radiology, Division of Nuclear Medicine, Weill Cornell Medical College, New York, NY, USA; ^4^Department of Pathology, Weill Cornell Medical College, New York, NY, USA

**Keywords:** prostate cancer, radioimmunotherapy, myelotoxicity, prostate-specific membrane antigen, monoclonal antibody

## Abstract

Radioimmunotherapy (RIT) has demonstrated efficacy with acceptable toxicity leading to approval in non-Hodgkin’s lymphoma, but has been slower to develop for the treatment of advanced solid tumors. Prostate cancer (PC) represents a good candidate for RIT based upon high exposure to circulating antibodies at common disease sites with a specific, highly expressed cell-surface antigen of prostate-specific membrane antigen. Four phase I and II trials utilizing ^177^Lu- or ^90^Y-J591 have been reported. Long-term toxicity and chemotherapy administration was analyzed. As expected, the only serious toxicity observed was myelosuppression. Grade 4 thrombocytopenia occurred in 33.3% without significant hemorrhage and grade 4 neutropenia occurred in 17.3% with 0.07% febrile neutropenia. Nearly all subjects (97.3%) recovered to grade 0 or 1 platelets and all had complete neutrophil recovery. The majority (81.3%) received chemotherapy at any time, with 61.3% receiving chemotherapy following RIT. Ten subjects underwent bone marrow biopsies at some point in their disease course following RIT for low counts; all had diffuse PC infiltration without evidence of myelodysplasia or leukemia. As expected, myelosuppression occurs following therapeutic doses of RIT for men with metastatic castration-resistant PC. However, toxicity is predictable and self-limited, with the majority of patients who do not refuse able to receive cytotoxic chemotherapy following RIT.

## Introduction

It is estimated that in year 2013, approximately 238,590 men will be diagnosed and 29,720 will die due to prostate cancer (PC) in the United States (1). Despite the effectiveness of hormone therapy, every patient with metastatic disease is currently incurable and those who live long enough eventually progress to castration-resistant prostate cancer (CRPC) (2).

Radioimmunotherapy (RIT), using specific monoclonal antibodies (mAbs) or fragments which are radiolabeled (most typically with beta-emitting particles), has proven quite effective in the treatment of non-Hodgkin lymphoma (NHL) alone or in combination with chemotherapy (3–5). RIT for solid tumors has posed a more difficult challenge for a number of biologic, technical, and practical reasons (6). In the last decade, significant progress has been made in the development of RIT for solid tumors. As with any therapy, emphasis is laid on balancing toxicity and therapeutic effects.

Metastatic PC is a good candidate for RIT, because it is radio-responsive and typically develops as small-volume metastatic sites of disease in marrow and lymph nodes that receive high levels of circulating antibody. Several clinical trials have focused on or included subjects with PC (7–16). Importantly, unlike some other solid tumors, a well-established, specific cell-surface antigen has been identified: prostate-specific membrane antigen (PSMA) (17–19). PSMA is an ideal target as it is expressed by nearly all PCs and is not secreted (17, 19). The expression levels progressively increase in more poorly differentiated, metastatic, and castration-resistant cancers (17, 20).

J591 is a deimmunized mAb which specifically binds with high affinity to the extracellular domain of PSMA (21, 22). In addition, the PSMA-J591 antibody complex is internalized thereby delivering any radionuclide or drug conjugated to the antibody to the interior of the targeted cancer cells (23). We have performed and reported 4 clinical trials using ^177^Lu and ^90^Y labeled J591 (24–28) (Table 1). Based on imaging studies, we have shown that J591 is able to sensitively and specifically target sites of metastatic PC in both bone and soft tissue (28). As often seen with RIT, radiolabeled-J591 was well-tolerated and serious toxicity was confined to predictable, reversible myelosuppression (24, 25, 27, 28). The preliminary efficacy data from initial phase I studies has been confirmed in a phase II clinical trial of single-dose ^177^Lu-J591 (28).

**Table 1:** **Radiolabeled-J591 clinical studies summary**.

Clinical trial	Agent used	Number of subjects	Cumulative dose (mCi/m^2^)	Dosing schedule
Phase I dose-escalation study using ^90^Y-J591 (24)	^111^In-J591 and ^90^Y-J591	29	5–20	^111^In-J591 for imaging followed by a single ^90^Y-J591 infusion*
Phase I dose-escalation study using ^177^Lu-J591 (25)	^177^Lu-J591	35	10–75	Single ^177^Lu-J591 infusion*
Phase II single dose trial (28)	^177^Lu-J591	47	65–70	Single ^177^Lu-J591 infusion^†^
Phase I fractionated dose trial (27)	^177^Lu-J591	39	40–90	^177^Lu-J591 infusion followed in 2 weeks by another at the same dose as first infusion

*Selected subjects with blood count recovery and lack of prostate cancer progression were eligible to receive additional infusions of ^177^Lu-J591.

*^†^An expansion cohort underwent imaging with ^111^In-J591 prior to treatment with ^177^Lu-J591*.

Several RIT studies have demonstrated that, in the absence of bone marrow or hematopoietic stem cell support, radiation-induced myelotoxicity is the dose-limiting toxicity (DLT) (29, 30). Hematologic toxicity is the biggest challenge faced by all radioimmunotherapeutic agents. The manifestations of myelotoxicity may be related to the pretreatment peripheral blood cell counts and bone marrow reserve, which may have been compromised by prior therapies (29, 31). While the vast majority of patients have spontaneous recovery of blood counts, some worry about the ability to deliver subsequent cytotoxic chemotherapy if needed clinically. The development of secondary myelodysplastic syndrome (MDS) or acute myelogenous leukemia (AML) has been reported (32) and has been estimated to be approximately 2–5% (33). Two studies have attempted to comprehensively evaluate the risk for MDS and AML following RIT with either ^90^Y-ibritumomab or ^131^I-tositumomab. These retrospective analyses of large numbers of patients both showed that RIT did not demonstrate a higher risk for MDS in comparison with similar patient populations treated with multiple chemotherapies alone (34, 35).

Long-term outcomes have rarely been reported with solid tumor RIT. Here, we report the long-term toxicity data in patients treated with radiolabeled-J591, including bone marrow recovery and ability to deliver cytotoxic chemotherapy.

## Materials and Methods

### Patient population

For each study, eligible patients had a prior histologic diagnosis of PC with evidence progressive metastatic disease as defined by a serum PSA and/or radiologic studies including bone scan, computed axial tomography, and/or magnetic resonance imaging despite castrate levels of serum testosterone (i.e., progressive metastatic CRPC) (24, 25, 27, 28). Patients were required to have platelet count of ≥150,000/mm^3^, hemoglobin ≥ 10, and neutrophil count of ≥2,000/mm^3^. Prior radiation to>25% of the skeleton and systemic beta-emitting radioisotope therapy (e.g., ^89^Sr or ^153^Sm) was exclusionary. All studies were approved by the Institutional Review Boards of participating institutions and registered on clinicaltrials.gov; all subjects provided written informed consent.

### Radiolabeled antibodies

Clinical-grade J591 mAb was covalently linked to the chelating agent, 1,4,7,10-tetraazacyclododecane-1,4,7,10-tetraacetic acid (DOTA) (21). The DOTA-J591 mAb was then radiolabeled with ^90^Y or ^177^Lu by incubation at 45°C (±2°C) for 45 min in the presence of an ammonium acetate buffer (pH 7.0). The final radiolabeled drug product was purified (when necessary) and filter sterilized before administration into patients as previously described (24, 25). Radiochemical purity was ≥97% at all cases as confirmed by instant thin layer chromatography. The immunoreactive fraction was always found>0.7 when tested in PSMA^+^-LNCaP cells.

### Treatment and follow-up

Subjects received single or multiple doses of radiolabeled-J591 intravenously at a rate not to exceed 5 mg/min per the particular protocol on which they were enrolled. Subjects were observed for a minimum of 12 weeks after their last dose of radiolabeled-J591 and those patients with stable or responding disease were observed until disease progression. Routine clinical and laboratory assessments (including metabolic profile, PSA, and testosterone) were performed at defined intervals. Complete blood count and platelet counts were monitored at least weekly, with more frequent monitoring with the onset of grade 3 thrombocytopenia or neutropenia. For most studies, white blood cell growth factors were allowed at the discretion of the investigator (not allowed in the fractionated dose-escalation study), and transfusions of platelets were also given at the discretion of the investigator. Following IRB approval, the number of previous therapies, the toxicity seen with radiolabeled-J591, the nature of subsequent therapies, and overall survival (OS) data were obtained by physician interview, medical record review, and post-treatment long-term follow-up with patients, families, or other physicians.

## Results

Between October 2000 and August 2012, 150 subjects with metastatic CRPC received radiolabeled-J591. One hundred and twenty-one patients received ^177^Lu-J591 at total doses of 20–90 mCi/m^2^ and 29 patients received ^90^Y-J591 at 5–20 mCi/m^2^. Patient baseline demographics are displayed in Table 2.

**Table 2:** **Baseline characteristics**.

Study	Total	^90^Y-J591	^177^Lu-J591
Patients, *n* (%)	150 (100)	29 (19.3)	121 (80.7)
Age, years (range)	70.7 (44.5–96)	70 (48–84.7)	71 (44.5–96)
Prior radiation to any location, *n* (%)	77 (51.3)	12 (41.4)	65 (53.7)
Prior radiation to prostate/prostate bed, *n* (%)	62 (41.3)	10 (34.5)	52 (42.9)
Cytotoxic chemotherapy prior to radiolabeled-J591, *n* (%)	67 (44.6)	14 (48.2)	53 (43.8)
Both chemotherapy and radiation prior to radiolabeled-J591, *n* (%)	35 (23.3)	4 (13.8)	31 (25.6)
Bony metastases, *n* (%)	129 (86)	20 (69)	109 (90.1)
Pulmonary metastases, *n* (%)	23 (15.3)	2 (6.9)	21 (17.4)
Hepatic metastasis, *n* (%)	12 (8)	1 (3.5)	11 (9.1)
Lymph node metastasis, *n* (%)	81 (54)	14 (48.3)	67 (55.4)

### Acute toxicity

Acute toxicity has previously been reported separately for each study (24, 25, 27, 28). Briefly, without pre-medication, 20.7% experienced transient grade 1 infusion reactions. Fourteen percent experienced transient low-grade transaminitis which returned to baseline in 100%. Platelet count decline was generally seen 2–3 weeks post infusion with platelet nadir occurring at 4–5 weeks after administration followed by a recovery phase. Grade 4 thrombocytopenia occurred in 33.3% patients without any significant hemorrhage. Thirty-five patients (23.3%) received platelet transfusion, 5 (17.2%) following ^90^Y-J591 and 30 (24.8%) following ^177^Lu-J591. Neutrophil decline typically occurred in parallel with thrombocytopenia, with nadir similarly 4–5 weeks following treatment. 17.3% experienced grade 4 neutropenia. One subject experienced grade 3 febrile neutropenia. Seventeen (11.3%) patients received granulocyte growth factor.

### Hematologic recovery

All subjects experienced improvement in blood counts following RIT induced nadir. Ninety-two percent had complete recovery of platelet counts to grade 0 (i.e., platelet count of at least 150,000/mcL) and 100% experienced recovery of neutrophil count to Gr 0 (i.e., ANC of at least 2000/mm^3^). Eight subjects (5.3%) recovered to grade 1 thrombocytopenia (platelet counts ranging from 99,000 to 140,000). Four subjects died of progressive PC prior to platelet recovery from nadir.

**Figure 1:**
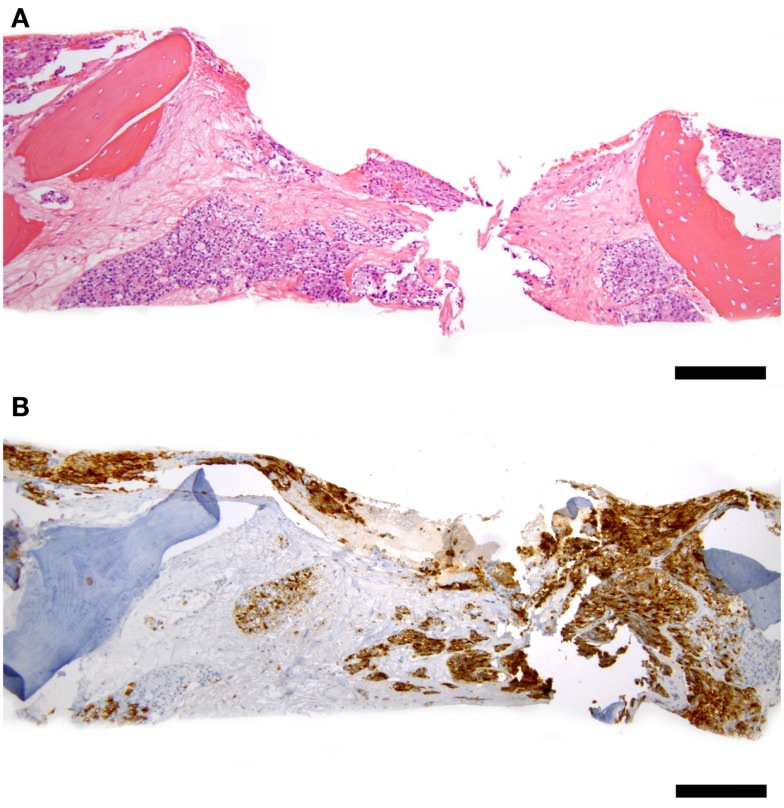
**Representative bone marrow biopsy of a patient with progressive prostate cancer and decreasing blood counts 3 years after ^177^Lu-J591 radioimmunotherapy, count recovery, and several subsequent therapies including chemotherapy**. **(A)** Hematoxylin and eosin stain at 100× total magnification low power view of bone marrow with intertrabecular marrow space entirely replaced by metastatic prostate cancer cells. **(B)** PSMA – 100× total magnification low power view of same field of bone marrow replaced by tumor showing PSMA-positivity in tumor cells (brown staining); scale bars = 200 microns. Cytogenetic studies revealed normal 46, XY male karyotype; normal bone marrow biopsy control not shown.

Some patients experienced full or partial platelet count recovery followed by subsequent decline. All were associated with evidence of simultaneous PC progression. Ten underwent bone marrow aspiration and biopsy confirming PC infiltration overtaking bone marrow (Figure 1). No evidence of MDS or leukemia was discovered.

The hematologic toxicity seen with radiolabeled-J591 is a known consequence of RIT. However, patient disease and previous treatment status can also contribute to amplification of such effects. 51.3% of patients treated with radiolabeled-J591 had history of previous radiation treatment, 44.6% had prior cytotoxic chemotherapy, and 23.3% had both prior chemotherapy and radiation (Table 2).

Grade 4 thrombocytopenia occurred in 40.3% (27/67) of patients with prior chemotherapy as compared to 27.7% (23/83) in the no-prior chemotherapy group (*p* = 0.10); 25.4% (17/67) with prior chemotherapy received platelet transfusions versus 21.7% (18/83) in the remaining patients (*p* = 0.59). Grade 4 neutropenia occurred in 17.9% (12/67) with chemotherapy prior to radiolabeled-J591 treatment versus 19.3% (16/83) in those who had no previous chemotherapy (*p* = 0.83) (Table 3).

**Table 3:** **Hematologic toxicity by prior therapy**.

	Prior chemotherapy (*n* = 67)	No-prior chemotherapy (*n* = 83)	Prior radiation (*n* = 77)	No-prior radiation (*n* = 73)	Prior chemotherapy and radiation (*n* = 35)	Neither prior exposure (*n* = 41)
Grade 4 thrombocytopenia, *n* (%)	27 (40.3)	23 (27.7)	26 (33.8)	24 (32.9)	16 (45.7)	13 (31.7)
Platelet transfusion, *n* (%)	17 (25.4)	18 (21.7)	19 (24.7)	16 (21.9)	11 (31.4)	10 (24.4)
Grade 4 neutropenia, *n* (%)	12 (17.9)	16 (19.3)	15 (19.5)	13 (17.8)	7 (20)	8 (19.5)

*No comparison was statistically significant (p > 0.05)*.

In those who had received prior radiation, grade 4 thrombocytopenia occurred in 33.8% (26/77) as compared to 32.9% (24/73) in the rest of patients (*p* = 0.90); 24.7% (19/77) with prior radiation treatment had platelet transfusions versus 21.9% (16/73) in no previous radiation therapy group (*p* = 0.69). Grade 4 neutropenia occurred in 19.5% (15/77) with pre radiolabeled-J591 radiation treatment versus 17.8% (13/73) in those who had no previous radiation therapy (*p* = 0.79).

In those who had received both prior chemotherapy and radiation, 45.7% (16/35) had Grade 4 thrombocytopenia versus 31.7% (13/41) with neither prior exposure (*p* = 0.21); 31.4% (11/35) received platelet transfusion with prior chemotherapy and radiation versus 24.4% (10/41) with neither prior exposure (*p* = 0.49) (Table 3).

### Therapies administered after radiolabeled-J591

81.3% of patients (122/150) received cytotoxic chemotherapy either prior to or after radiolabeled-J591. In review of long-term follow-up data, 92 (61.3%) received cytotoxic chemotherapy post radiolabeled-J591 infusion. Sixty-one percent (56/92) patients who received chemotherapy post RIT were chemo-naive prior to treatment and 39% (36/92) received chemotherapy both before and after RIT. 13.3% (20/150) of patients received investigational treatment following RIT, 24.6% (37/150) had no active treatment, 13.3% (20/150) were deemed chemotherapy eligible by their physicians, but declined subsequent chemotherapy and 12% (18/150) enrolled in hospice or died before starting a new treatment.

## Discussion

Prostate cancer offers a model for the investigation and development of RIT in solid tumors, given the restricted, high-level of expression of PSMA and the availability of a specific, well-tolerated mAb (J591). As myelosuppression and subsequent bone marrow recovery as well as the theoretical inability to tolerate subsequent cytotoxic chemotherapy are potential issues with RIT, we analyzed our long-term results with radiolabeled-J591. It should be noted that this analysis included radiolabeled-J591 administered across a number of different cumulative doses in phase I and II studies, including a prospective fractionated dose schedule (Table 1).

The vast majority of subjects who received radiolabeled-J591 had complete (i.e., grade 0) or near-complete (grade 1) recovery of neutrophil (100%) and platelet (97.3%) counts. Those that did not experience complete recovery or who experienced subsequent decline in blood counts had concomitant progression of PC. Those with bone marrow biopsies at subsequent count decline had diffuse PC marrow infiltration and none had evidence of MDS or leukemia. As some have proposed that prior treatment might influence subsequent bone marrow reserve and the ability to tolerate RIT, (31) we analyzed by previous exposure to chemotherapy and/or radiation. There was a trend for more grade 4 thrombocytopenia in those with both prior chemotherapy and radiation, but no clear differences in those who had received either previous chemotherapy or radiation.

Because of the myelosuppression observed in patients treated with radiolabeled-J591, a further concern is whether patients can tolerate subsequent therapeutic interventions if needed for disease progression after RIT. The available data suggest that patients treated with radiolabeled-J591 can tolerate subsequent therapies. The majority (92 of 150, 61.3%) of patients received cytotoxic chemotherapy after radiolabeled-J591, including docetaxel and cabazitaxel. Although two of the four studies were completed prior to the approval of docetaxel for metastatic CRPC, 95 of 150 (63.3%) received docetaxel at any time, a favorable number compared to the estimated third of men with metastatic CRPC ever receiving docetaxel according to a survey of urologists and oncologists in the era prior to the approval of newer agents such as Sipuleucel-T, Abiraterone, and Enzalutamide. Another concern might be a theoretic possibility of poorer chemotherapy tolerance in the setting of prior RIT. While this cannot be completely addressed in a retrospective study, patients who received docetaxel at any time following radiolabeled-J591 received a median of 23 weeks of chemotherapy (range 10–68) (i.e., 7–8 cycles) and those who received cabazitaxel received a median of 18 weeks (range 17–36) (i.e., 5–6 cycles), both within range of general community standards.

One of the most serious concerns about RIT is its potential to lead to secondary malignancies, particularly MDS/AML. The long-term data following RIT of solid tumors is limited, in part for the practical reason of populations that have been studied (generally late stage, refractory cancers). In this study, subjects were treated over a 12-year period with median time from treatment of 7.8 years (range 0.7–12.7); censored for loss to follow-up or death, median follow-up for this analysis was 16.6 months (range 0.5–133.9). The estimated median survival for the population based upon nomogram analysis is 15 months (median Halabi score 145, range 62–196) (36). As solid tumor RIT moves earlier in the disease process and as median survival for advanced solid tumors increases with more successful therapy, long-term toxicity follow-up is needed. More data exist following RIT for NHL. With a median 10-year follow-up, Kaminski et al. reported a single case of MDS out of 76 previously untreated patients who received a single treatment with ^131^I-tositumomab (37). Czuczman et al. reviewed the records of 746 patients treated with ^90^Y-ibritumomab tiuxetan with a median follow-up of 4.5 years and found a total of 2.5% of patients developed MDS at a median of nearly 2 years following RIT (35). This corresponded to an annualized MDS rate of 0.7% per year following RIT. The expected annual rate of treatment-related MDS for NHL patients receiving systemic therapy alone or in combination with rituximab is approximately 1% per year (38, 39). Higher doses of therapy may pose greater risk. Recently, Guidetti et al. published prospective data on development of MDS/AML in NHL patients receiving myeloablative doses of ^90^Y-Ibritumomab tiuxetan. Among 52 patients with a median follow-up of 49 months, the 5-year cumulative incidence of MDS/AML was 8.29% (40). This incidence is significantly higher than previously reported with lower doses, but the studied population consisted of subjects receiving significantly higher doses of RIT than used for solid tumors and a matched-pair analysis of patients receiving myeloablative chemotherapy conditioning instead of RIT revealed a similar 8.05% 5-year cumulative incidence of MDS/AML.

Taken together, although there have been few prospective studies designed specifically to assess the risk of development of MDS/AML following RIT for NHL, with significant follow-up, the use of RIT does not appear to significantly increase the risk of secondary MDS above the risk of chemotherapy alone in patients with NHL. Should cases of MDS or leukemia be discovered with radiolabeled-J591 or other RIT for PC, it will be important to understand that as in NHL, additional therapies may be associated with MDS/AML and there is a *de novo* incidence in an elderly male population (35, 41, 42).

## Conclusion

Systemic targeted radiation with RIT has therapeutic promise in advanced solid tumors, in particular for radiosensitive tumors such as PC with a selective and specific cell-surface antigen such as PSMA and an available antigen-specific, non-immunogenic mAb such as J591. Using beta-emitting radionuclides at therapeutic doses, myelosuppression is expected. However, toxicity is predictable and usually self-limited, with the majority of patients who do not refuse able to receive chemotherapy. As the use of RIT becomes more attractive across diseases with the development of newer, more specific mAbs or peptides, studies examining long-term toxicity are warranted.

## Conflict of Interest Statement

Neil H. Bander is an inventor on patents that are assigned to Cornell Research Foundation (“CRF”) for the J591 antibody described in this article. Dr. Bander is a paid consultant to and owns stock in BZL Biologics, the company to which the patents were licensed by CRF for further research and development. The other co-authors declare that the research was conducted in the absence of any commercial or financial relationships that could be construed as a potential conflict of interest.
